# Self-esteem associated with the use of social networks in university students

**DOI:** 10.1093/eurpub/ckac131.255

**Published:** 2022-10-25

**Authors:** JE Villegas Dominguez, BL Muñoz Bautista, EA Gonzalez David, R Vazquez Garate, S Uscanga Alcantara, JJ Pavan Gallardo, C Reyes Hernandez, AJ Cruz Garduza, FG Marquez Celedonio

**Affiliations:** 1 Facultad de Medicina, Campus Veracruz, Universidad Veracruzana, Veracruz, Mexico; 2 Facultad de Medicina, Universidad el Valle de Mexico, Veracruz, Mexico

## Abstract

**Introduction:**

Currently, the consequences of social networks on the self-esteem of users have been demonstrated in dissemination sources, and can generate problems in their mental and physical health.

**Objective:**

To determine the association between self-esteem and addiction to social networks in university students.

**Methods:**

A cross-sectional, prospective study was carried out between January and April 2022, including male and female university students, aged between 18 and 26 years, excluding participants with already diagnosed psychological disorder, such as depression, anxiety and body dysmorphia. A survey was applied through Google Forms, including the Rosenberg self-esteem scale and the social network addiction test (Cronbach’s alpha 0.91). SPSS v22 software was used for data analysis, X2 test with Odds Ratio (OR) and 95% confidence interval (95%CI), assigning statistical significance with p < 0.05.

**Results:**

A total of 407 students were included, with moderate social network addiction in 57.4% and severe in 8.1%, with low self-esteem in 22.1% and moderate in 50.6%. Values of p < 0.05 were obtained for low self-esteem (OR/95%CI) when the most used social network is WhatsApp (0.5/0.2-0.8) and Instagram (1.8/108-3.2), as well as when having a number of followers between 1501-2000 (0.2/0.1-0.7) and 2501-3000 (10.8/1.1-106). Regarding network addiction, studying a humanities degree showed OR of 12(1.6-88.1). The rest of social networks such as Facebook, tik-tok, twitter, youtube, among others, showed values of p > 0.05 for both addiction to social networks and low self-esteem.

**Conclusions:**

Instagram is identified as a social network that facilitates the presence of low self-esteem in its users, while addiction to social networks is not associated with self-esteem. The number of followers is an associated factor for low self-esteem, and may be preventive or risky according to their total.

**Key messages:**

• We must propose the legislation of social networks, requesting the guarantee of studies for the algorithms that compose them in order to avoid harm to users from them.

• Work with students to have mental health and emotional intelligence should be one of the aspects to be covered by universities, as part of the comprehensive care for students.

